# Incorporation of an ultrasound and model guided permissible region improves quantitative source recovery in bioluminescence tomography

**DOI:** 10.1364/BOE.9.001360

**Published:** 2018-02-27

**Authors:** Baptiste Jayet, Stephen P. Morgan, Hamid Dehghani

**Affiliations:** 1 Optics and Photonics Research Group, Faculty of Engineering, University of Nottingham, University Park, Nottingham, NG7 2RD, UK; 2 School of Computer Science, University of Birmingham, Edgbaston, Birmingham, B15 2TT, UK

**Keywords:** (110.0113) Imaging through turbid media, (170.3010) Image reconstruction techniques, (170.3880) Medical and biological imaging, (170.7170) Ultrasound, (100.3190) Inverse problems

## Abstract

Bioluminescence imaging has shown great potential for studying and monitoring disease progression in small animal pre-clinical imaging. However, absolute bioluminescence source recovery through tomographic multi-wavelength measurements is often hindered through the lack of quantitative accuracy and suffers from both poor localisation and quantitative recovery. In this work a method to incorporate a permissible region strategy through not only *a priori* location (permissible region) but also based on a model of light propagation and hence light sensitivity is developed and tested using both simulations and experimental data. Reconstructions on two different numerical models (a simple slab, and the digital version of a heterogeneous mouse) show an improvement of localisation and recovery of intensity (around 25% for the slab model and around 10% for the digital mouse model). This strategy is also used with experimental data from a phantom gel, which demonstrated an improved recovered tomographic image.

## 1. Introduction

Bioluminescence imaging (BLI), as applied in molecular imaging, has gained interest over the last two decades. Coding genes for bioluminescent protein provides specific tools to study a wide variety of biological processes directly *in vivo*, such as detection of cancer cells [1] and evaluation of treatment [2, 3]; imaging of T cell migration [3]; or tracking the delivery of therapeutic genes in tumours [3]. In addition to this broad range of applications, the interest in such methods resides in the fact that the biological interactions are monitored directly within a living organism, which provides a better understanding of the complex interactions occurring *in vivo*, thus allowing each animal to act as its own control. BLI also offers the possibility of 3D tomographic reconstruction of the light sources inside the living sample. Due to the absence of external light sources, contrarily to techniques based on fluorescence, and localised sparse distribution of light sources, BLI has very little parasitic background light. This makes BLI very sensitive, provided that the emitted light is intense enough to be detected at the external boundary using highly sensitive cameras, such as cooled Charged Coupled Devices (CCD). As objects of studies are living organisms, which are strongly scattering, light levels in BLI experiments, typically in the 500 nm − 650 nm range, are low and, as a result, only sources at the surface or very shallow within the sample (less than a few mm) can be localised and quantified without difficulty. However, for deeper sources, tomographic reconstruction to obtain an accurate localisation and intensity is difficult.

To perform a bioluminesence tomographic reconstruction (BLT), a model (discrete and finite or analytical) is needed to allow model-based optimisation and parameter recovery. Numerical models typically consist of a set of nodes that describes the geometry and the optical properties (absorption, scattering and refractive index) of the studied sample. To each node is also associated a bioluminescence source intensity that can be written as a vector **x**. The image taken by the CCD of the light distribution constitutes surface data that can be written as a vector **y**. Using light propagation models in complex media [4, 5], it is possible to relate **x** and **y** with a linear system of equations that can be written as **Jx=y**, where the matrix **J** contains the physics of the light propagation through the studied sample, given an accurate knowledge of its optical properties as a function of **x**. **J** is usually referred to as the Jacobian (or weight or sensitivity) matrix and the aim of a BLT reconstruction is to find **x** knowing **J**, which is calculated or approximated; and **y**, which is given by experimental measurements. These problems are usually underdetermined since the number of measurements (typically around a few hundreds to a few thousands) is much lower than the number of unknowns (typically around a few tens of thousands). In addition, the strong correlations between measurements, mainly due to the strong light scattering by biological tissues, reinforce the underdetermined nature of the system.

Various strategies have been developed to improve the accuracy of BLT reconstructions, consisting of either experimental or computational methods, or a combination. Experimental methods include, for example, the use of spectrally resolved data to improve depth accuracy and error in intensity [6]. Computational strategies can act on the model used (radiative transfer equation or diffuse approximation) [7, 8], take advantage of the usual sparsity of BLT sources [9–11], define a permissible region (PR) or a combination of these approaches. There are various permissible region strategies, for example, Cong *et al*. [12] which sets to 0 all the source terms outside a specific region and Feng *et al*. [13] which uses an iterative process to make the permissible region iteratively smaller. Finally, combination strategy can incorporate information from other imaging modalities such as MRI [14, 15], CT [15] or microCT [1, 2] to inform or adapt the numerical model.

These permissible region strategies have shown to improve recovery of bioluminescent sources. However, the definition of the permissible region does not rely on specific criteria. The aim of this work is to propose a new way of defining a permissible region using characteristics of the model and information about the source provided by another imaging method: ultrasound imaging. Previous works on coupling BLT and ultrasound have mainly been concerned with investigating the effects of the acoustic wave on the light emission, through thermal effects [16] or through the modulation of the optical properties [17]. In this work, ultrasound is not used to modulate the light but simply as a guide for the definition of a PR for the reconstruction. In addition to using ultrasound, another novelty of this approach is to drive the PR definition using model based analysis. Using this approach, a more quantitatively accurate source recovery and a better 3D source localisation is demonstrated. This novel strategy is implemented for BLT reconstruction problem in NIRFAST [18, 19], which is an open source software package designed for optical molecular imaging in scattering media. Currently, NIRFAST uses a Compressive Sensing Conjugate Gradient (CSCG) approach [11] to find solutions to BLT problems without PR definition.

## 2. Theory

Typical permissible source region strategies limit the size of the reconstructed volume along only two dimensions as the depth of the source cannot be estimated using the CCD image. However, by using ultrasound imaging, it is possible to estimate the depth of the source to center the permissible region more accurately. The size of the permissible source region can be set by optimising the model for inversion. This is demonstrated to be crucial as small sensitivity values are greatly amplified by the inversion process as compared to high values resulting in amplified noise and loss of accuracy. Therefore, this work proposes to truncate the Jacobian describing the model into a sub-Jacobian with a smaller dynamic range retaining only the nodes relevant to the problem. This truncation is equivalent to assuming that the intensity of the light emitted by the nodes outside the truncated volume is 0. The definition of the permissible region for a specific dynamic range *d* is defined as:

1. The coordinates (*x*_0_, *y*_0_, *z*_0_) of the source are determined using ultrasound imaging.
2. The closest node to (*x*_0_, *y*_0_, *z*_0_) is chosen as the reference node, its sensitivity is *s*_0_
3. Given a model with *N* nodes (numbered 1 to *N*), the list of nodes of interest (NOI) is $\left\{i\in [1;N]\;:\;s_{i}\in \left[\frac{s_{0}}{\sqrt{d}};\;s_{0}\sqrt{d}\right]\right\}$, where *s_i_* is the sensitivity of node *i*. The (total) sensitivity on node *n* is then defined as $s_{n}=\sum_{j=1}^{M}J_{j,n}$ where *J*_*j*,*n*_ is the value of the Jacobian for the *j^th^* measurement on node *n*, and *M* is the number of measurements.

The list of NOI is then used to truncate **J**, which is then used together with the CSCG solver (detailed in [11]). By limiting the dynamic range of the system, the permissible region is confined in all three dimensions, especially along the z-axis, which corresponds to the depth of the source. This approach also reduces the number of degrees of freedom of the algorithm which yields a more accurate recovered intensity, while removing noise amplification of lower sensitivity nodes. This proposed ultrasound guided reconstruction assumes that the bioluminescent source has a contrast in ultrasound imaging or related techniques (*e. g*. elastography).

## 3. Methods

To investigate the performance of the proposed algorithm, two sets of studies are presented. This is firstly carried out using simulations, to demonstrate the benefits of the proposed reconstruction strategy, followed by an application of the proposed reconstruction strategy to real experimental data taken on a phantom with a commercial bioluminescent imaging system (IVIS system).

### 3.1. Simulation methodology

The simulation work was carried out on MATLAB™ using the NIRFAST software package. The simulations were performed in two steps:

1. Nodes of the studied mesh within the source region were defined as bioluminescent sources and the forward solver of NIRFAST was used to calculate the data that would typically be recorded during a real *in vivo* bioluminescent experiment using 5 different wavelengths in the red region of the optical spectrum (600 nm, 610 nm, 620 nm, 630 nm and 640 nm).
2. The calculated forward model data are then used as an input for reconstructions using three different methods:

The forward model data are corrupted with noise, which is assumed to be a normally distributed Gaussian noise with a typical level for BLT experiment (SNR = 20 dB). After the reconstruction, the recovered tomographic image by each method is compared visually to the expected image, which is known, as well as, using a number of metrics described in section 3.2. The reconstruction using each different method is run 100 times using different Gaussian noise data. From every reconstruction, the mean value and standard deviation of each defined metric has been calculated and presented.

**Fig. 1:**
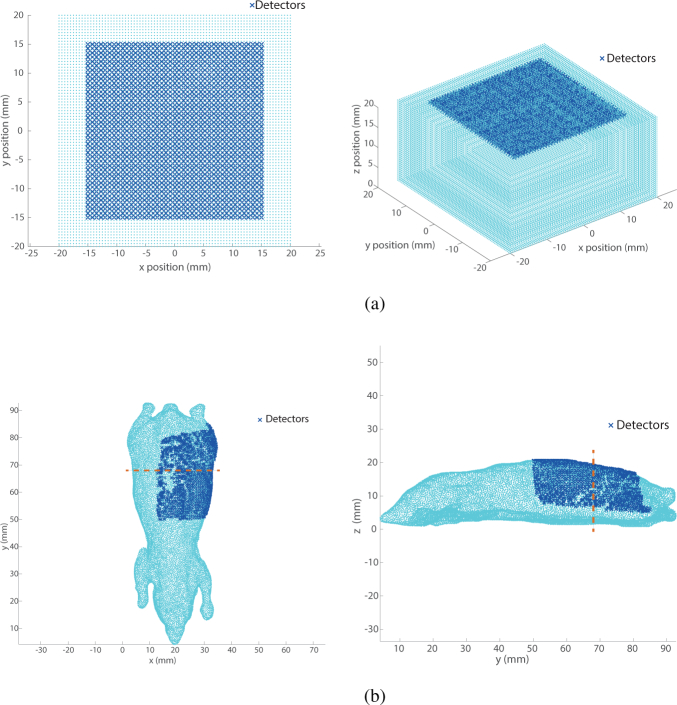
Geometry of the meshes used in the simulations. (a) Slab mesh, (b) Digimouse mesh. The dashed line represents the cross-section used in the visual representation of section 4.2.

This simulation work is carried out on two types of model. The first is a homogeneous slab-shaped mesh (see Fig. 1(a)

) with the same optical properties as muscle tissue [20], as shown in Table 2

**Table 1:** Reconstruction methods compared in this study.

Name	Description	Name in Figures
Whole mesh reconstruction	Reconstruction using all the nodes of the model	Whole Mesh
Large Permissible region re-construction	Reconstruction using a large permissible region limited only on two dimensions. No depth limit	Large PR
US guided and model driven reconstruction	Reconstruction using a permissible region with the centre defined using ultrasound imaging and the size determined by the dynamic range of the sensitivity matrix	US Guided PR

**Table 2:** Geometrical and optical properties of the slab mesh.

Dimension (*x* × *y* × *z*)	40 mm × 40 mm × 20 mm
Node distance	0.5 mm
Total number of nodes	58509
Chromophore list	Water, Oxyhaemoglobin and Deoxyhaemoglobin
Refractive index	1.33
Scatter amplitude	0.14
Scatter power	2.82

. A set of detectors is positioned on the top side *z* = 20 mm. The *x* and *y* positions of the detectors correspond to a 30 mm × 30 mm square grid having a 0.75 mm pitch. This results in a total of 1681 detectors. The second mesh is the Digimouse model [21, 22], which is a digital version of a mouse with organ segmentation. The optical properties of the different organs have been set according to [20]. On the left back of the animal, a set of 1324 detector points is placed to record the forward model data as shown by Fig. 1(b).

### 3.2. Metrics

The performance of the proposed ultrasound guided reconstruction is evaluated using three metrics: the Volume Ratio (VR), the DICE similarity coefficient (DICE) and the Mean Square Error (MSE) [23]:

$$VR=\frac{\left|ROI\right|}{\left|tROI\right|}\;DICE=\frac{2\left|ROI\cap tROI\right|}{\left|ROI\right|+\left|tROI\right|}\;MSE=\frac{1}{N}\sum_{i=1}^{N}{\left(x_{i}-x_{0i}\right)}^{2}$$

Where *ROI* represents the reconstructed source nodes, *tROI* are the true source nodes, *x_i_* and *x*_0_*_i_* are the intensity values on the reconstructed nodes and the true image nodes respectively. The |·| operator indicates the physical volume occupied by the corresponding set of nodes. The reconstructed source nodes are identified as the nodes having a value greater than the median nodal value. This ensures that nodes with very high reconstructed intensities do not influence the threshold, which would occur if it was defined as half the maximum. However, since the modelled source is small – only a few nodes compared to the ten thousands of the mesh – there are a large number of nodes with a 0 value which will bias the median. Therefore the median is calculated on a set of nodes that have a value greater than 1% of the expected value. In addition to these metrics, the reconstruction methods are also visually evaluated and compared.

The VR compares the reconstructed source volume to the true source volume, therefore it should be as close to 1 as possible. However, a value of 1 does not guarantee a good reconstruction as the reconstructed source could be of the same volume but at a different position. The DICE coefficient complements VR by comparing the volume of the intersection between the reconstructed and the true source and, similar as the value of the VR, should ideally be equal to 1. These first two metrics evaluate only geometrical similarities, hence the use of the MSE as a third metric to evaluate the accuracy of reconstruction in terms of absolute recovered intensity.

### 3.3. Phantom preparation and experiments

For the phantom experiment, a 70 mm × 40 mm × 20 mm (*x* × *y* × *z*) slab of agar gel was used. It had embedded polystyrene microspheres to have reduced scattering coefficient around $\mu_{s}^{\prime }=5\;{\text{cm}}^{-1}$ (lower than the usual value for biological tissue). No absorbers were added so the absorption is the natural absorption of water, agar and polystyrene (*µ_a_* ≈ 0.0004 cm^−1^). In this phantom, a small hole at *z* = 5 mm has been made in order to embed a small light source. The source is a plastic tube made of Fluorinated Ethylene Propylene (FEP) filled with a chemiluminescent solution having a peak emission wavelength of 640 nm (*FWHM* = 60 nm). The tube has an inner diameter of 2 mm and a length of 5 mm.

After placing the light source in the gel, it is imaged using an IVIS system at 5 wavelengths (620 nm, 640 nm, 660 nm, 680 nm and 700 nm). Since the source has been placed at *z* = 5 mm, images have been taken on both sides (*z* = 0 mm and *z* = 20 mm) to have data for two different depths. Finally, ultrasound images of the phantom have been taken to have a better localisation of the light source. This was done using a Verasonics system (Vantage 128) equipped with an ultrasound probe (Verasonics L11-4v) working at a central frequency of 6.25 MHz.

Finally, the data acquired by the IVIS system are used together with a numeric model for image recovery. The model used for the reconstruction of the experimental data has been generated using NIRFAST meshing algorithm [19]. It is a slab mesh with the same physical dimension as the gel: 70 mm × 40 mm × 20 mm. A 50 mm × 30 mm grid of detector points with a 1 mm pitch has been placed on the surface *z* = 20 mm (total 1581 detector points). Then data recorded with the system are mapped onto this set of detectors before being used for the reconstruction.

## 4. Results

### 4.1. Simulation on slab model

The first simulated model is a slab as represented in Fig. 1(a), with the geometrical properties detailed in Table 2. In bioluminescence imaging, when a light source is deep inside the tissue, usually deeper than 10 mm, the intensity and position is difficult to recover. Therefore, sources at depth of *d* = 15 mm and at position *x* = 0 and *y* = 0 are studied. In every simulation presented, the source is a cylinder with a radius of *r* = 1.5 mm and a length of *h* = 5 mm. The intensity of the source is set as arbitrary units of 10, which means that every node that is tagged as a source emits an intensity of 10. Therefore, it is expected that, in the perfect case, the reconstructed intensity should also have a value of 10 for each recovered source.

**Fig. 2:**
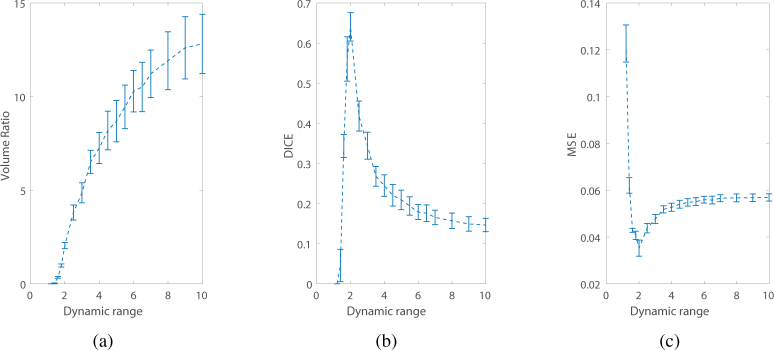
Metrics *vs*. dynamic range for the guided reconstruction of a source 15 mm deep in the slab mesh. (a)VR *vs*. dynamic range, (b)DICE *vs*. dynamic range, (c)MSE *vs*. dynamic range.

First, the quality of reconstruction *versus* the dynamic range of the Jacobian matrix is studied. Reconstructions of a source 15 mm deep in the sample using various values of the dynamic range have been performed. The variation of the defined metrics *vs*. the dynamic range are shown in Fig. 2

. The plots of the DICE and MSE show that the optimal values of the dynamic range lie between 1.5 and 3 (as a comparison the dynamic range of the sensitivity matrix for the entire model is around 5300). Indeed, if the dynamic range is too small, the permissible region will be small also, such that the nodes inside the true source are excluded from the reconstruction. Conversely, when the dynamic range is too large, too many nodes with different sensitivity are included resulting in a less stable and accurate result as shown in Fig. 2. This demonstrates that the dynamic range must not be chosen to be as small as possible since one has to make sure that the permissible region is large enough to contain the bioluminescent source. As the idea is to use ultrasound imaging to estimate the position of the source, its size can also be estimated using the same method.

**Fig. 3:**
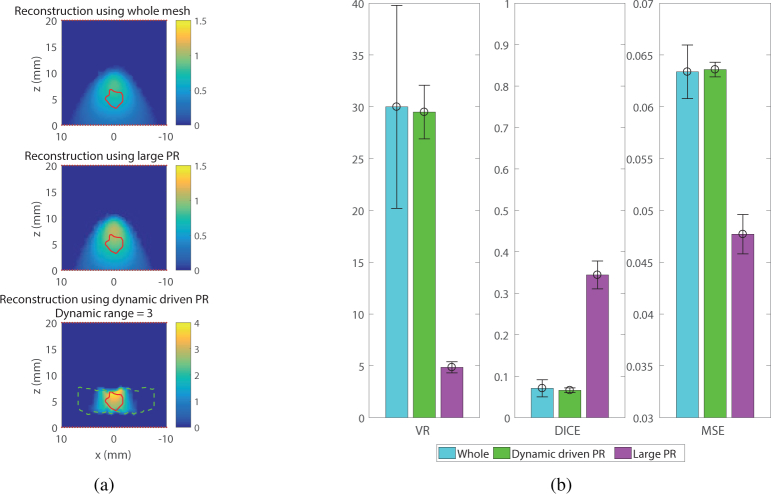
Reconstruction performances of a source 15 mm deep inside a slab mesh. (a) Visual example of the reconstruction: (Top) Whole mesh, (Middle) Large PR, (Bottom) US guided PR, (b) Metrics of three reconstruction methods.

In order to compare the model driven approach and the two other reconstruction methods (as described in Table 1), a value of 3 has been chosen for the dynamic range. This value, at the upper end of the optimal value, has been chosen to act as the worst case scenario where the optimal dynamic range cannot be determined. Indeed, in a real BLT experiment, the information about the source location are not known so the VR, DICE and MSE cannot be estimated. The reconstructions for one realisation of the noise using the three different methods is shown in Fig. 3(a)

. In this representation of the XZ plane at *y* = 0, the green dashed line is the limit of the permissible region and the red solid line is the limit of the true bioluminescent source (it is not perfectly circular because of the non-uniform distance between mesh nodes). It is demonstrated that the reconstructed intensity is better localised using the guided reconstruction, especially in the vertical axis. This was expected since the algorithm works only on a permissible region around the real position of the source that is restricted along the vertical dimension. The other, and more interesting point to notice, is the value of the reconstructed intensity. The bioluminescent intensity reconstructed with the guided algorithm is closer to the true value of the intensity than the intensity reconstructed using the whole mesh or the large permissible region. These absolute recovered intensities are quantified using the metrics as shown in Fig. 3(b).

In Fig. 3(b) each bar represents the value of each metric for each reconstruction method averaged over 100 different instances of noise added data. The standard deviation is shown in the corresponding error bar. Both the results for VR and DICE are closer to 1 using the guided reconstruction, which illustrate a more accurate localisation of the source. Moreover, the lower MSE reflects a better accuracy of the guided reconstruction approach. This first simulation on a simple model shows promising results, demonstrating that guiding the reconstruction is improving both the geometry and, more importantly, quantitative accuracy.

**Fig. 4:**
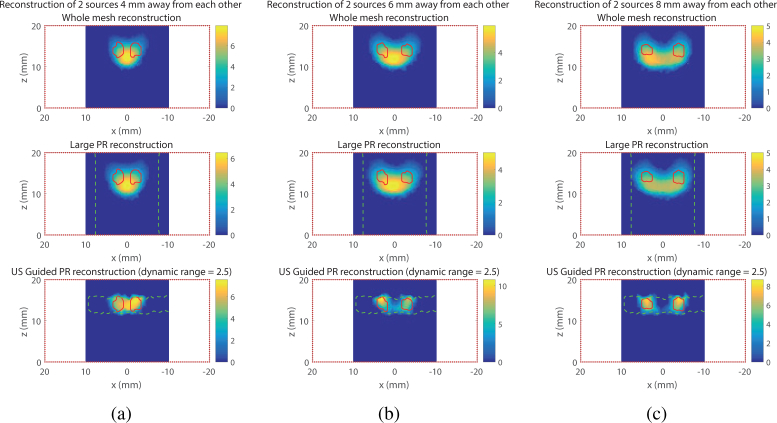
Visual example of the reconstruction of two 6 mm deep sources separated by different distances. (Top) Whole mesh, (Middle) Large PR, (Bottom) US Guided PR. (a) Sources separated by 4 mm, (b) Sources separated by 6 mm, (c) Sources separated by 8 mm

**Fig. 5:**
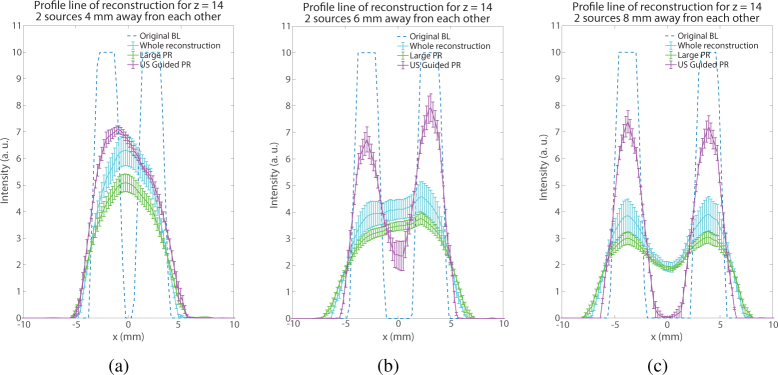
1D profile at *z* = 14 mm of the reconstruction of two 6 mm deep sources separated by (a) 4 mm, (b) 6 mm, (c) 8 mm.

The benefits of the proposed approach can also be seen with simulation at shallower depth, but with multiple sources. In this series of simulations on the slab model, two 6 mm-deep sources spaced by 4 mm, 6 mm or 8 mm have been placed in the model. Again, it is demonstrated by Fig. 4

that the guided reconstruction performs better especially in the 6 mm-spacing and 8 mm-spacing cases. It is particularly illustrated by the 1D profiles for *z* = 14 mm (position of the source on the *z*-axis) in Fig. 5

. The contrast of the two sources is much higher in both cases using the guided reconstruction than the other conventional methods.

### 4.2. Simulation on Digimouse model

Further investigations were carried out using a more realistic model: the Digimouse mesh represented on Fig. 1(b). This time, the source is a sphere with a radius of *r* = 1.5 mm, placed under the skin at a depth of either 7.2 mm or 9 mm. As before, the reconstructions are run 100 times using different realisations of Gaussian noise. The chosen dynamic range for the guided reconstruction is 3, whereas for comparison, the dynamic range of the sensitivity of the whole mesh is approximately 20000.

**Fig. 6:**
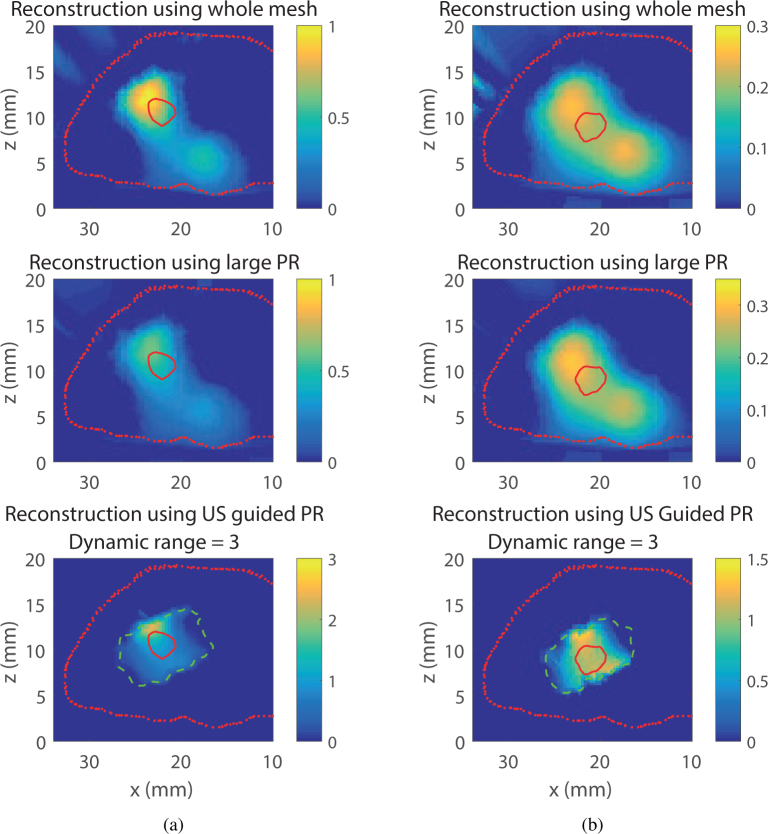
Visual comparison of three reconstruction method (Whole mesh (Top), Large PR (Middle), US Guided PR(Bottom)), for a source in the Digimouse mesh at two different depths. (a) Source depth = 7.2 mm, (b) Source at depth = 9 mm.

**Fig. 7:**
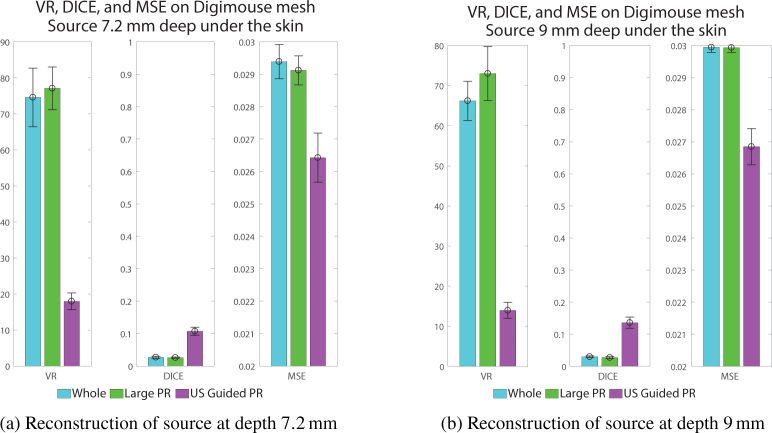
Metrics of three reconstruction methods for a source in the Digimouse mesh at two different depths. (a) Source depth = 7.2 mm, (b) Source at depth = 9 mm.

The three reconstruction methods for one realisation of the noise in the XZ plane situated at *y* = *y_S_* (where *y_S_* is the *y* coordinate of the center of the source, orange line on Fig. 1(b)) for the 7.2 mm deep source is shown in Fig. 6(a)

, whereas, Fig. 6(b) is the same for the 9 mm deep source. In these images, the red dotted line is the external outline of the model in the chosen plane, the green dashed line is the limit of the permissible region and the red solid line is the limit of the true bioluminescent source. Whether the source is 7.2 mm or 9 mm under the skin, the whole mesh reconstruction and the large permissible region reconstruction produce a large dispersed source that could even be interpreted as two separate sources. This is primarily due to the large dynamic range of sensitivity values which during the inversion process have resulted in "false-positive" cases of recovered sources. Conversely, the guided reconstruction offers a more localised recovered source, as the dyanmic range of the sensitivity values are limited through the model-based PR applications. Also, same as observed with the slab mesh, the reconstructed intensity is closer to the true intensity. This is confirmed by the value of the metrics showed in Fig. 7

, which shows a VR and DICE value closer to 1 as well as a lower MSE for the US guided reconstruction.

### 4.3. Phantom study

Finally, the proposed guided reconstruction has been compared with conventional reconstruction using the whole model on experimental data taken on a scattering phantom using a commercial BLT system (IVIS). In this experimental case, the intensity and optical properties of the source are not known accurately enough to be able to calculate the metrics. Therefore only qualitative visual comparison on the reconstructed images are provided and discussed.

**Fig. 8:**
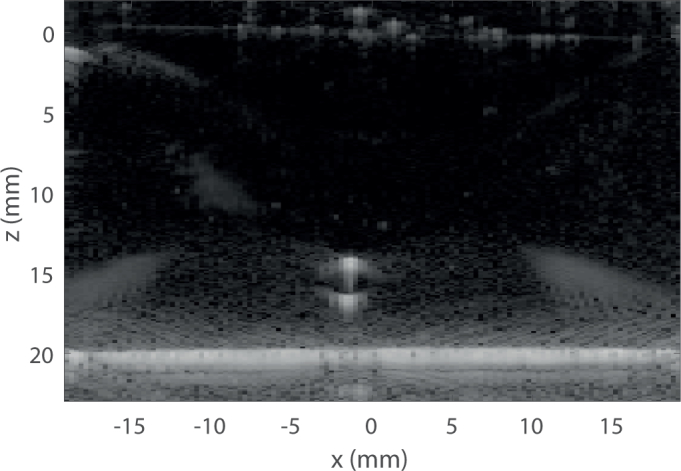
Ultrasound image of the experimental gel.

In addition to measuring optical bioluminescence data, the gel was also scanned using an ultrasound probe in order to obtain the position of the source. An XZ slice of the gel acquired using ultrasound imaging is represented in Fig. 8

. On this image, the vertical axis has been shifted so that the edge of the gel is positioned at *z* = 0. The chemiluminescent source produces an intense echo, mainly due to the plastic tube encapsulating the solution, which can be easily identified. The position of the center of the source is at *z* = 15.5 mm, therefore the depth used to generate the permissible region will be *z_PR_* = 4.5 mm or *z_PR_* = 15.5 mm depending on which set of data is used for reconstruction.

**Fig. 9:**
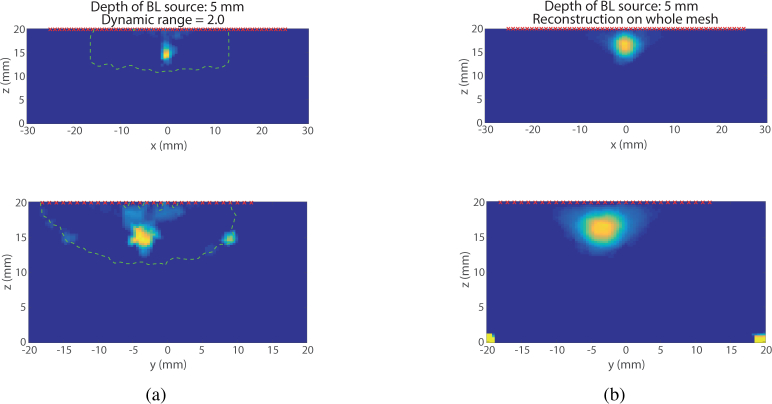
Visual comparison of two reconstruction methods (US Guided PR (a) and Whole mesh (b)) for a source 4.5 mm deep in the experimental phantom. (Top) XZ Plane, (Bottom) XY Plane.

In these reconstructions, a value of the dynamic range of 2 has been used for the guided reconstruction. The choice of a smaller dynamic range was motivated by the fact that the dynamic range of the whole Jacobian is smaller (510 against 5300 for the slab mesh and 20000 for the Digimouse mesh), due to a lower absorption coefficient (the gel is mostly water). The first set of data used for the reconstruction are the data acquired for a depth of 5 mm. Figure 9

shows the XZ plane (top) and YZ plane (bottom) for the reconstruction of the source using the guided method (9(a)) and using the whole mesh (9(b)). Since the absolute value of the source intensity is not known in these examples the recovered value is arbitrary and cannot be compared to a ground truth, whereas its size and location can. As in the previous example, the green dashed line is the limit of the permissible region. The red crosses correspond to the position of the detectors on the surface of the mesh. Both reconstructions have located the source at the correct position. Nevertheless, the source reconstructed using the guided reconstruction appears to have a size closer to the size of the true source as compared to the source reconstructed using the whole mesh.

The size of the reconstructed source has been measured in all cases. It has been estimated by taking the Full Width at Half Maximum (FWHM) of profiles along *x* and *y* axes, the results are compiled in Table 3

**Table 3:** Reconstructed source dimension.

	5 mm-deep source	15 mm-deep source
Original dimension	*dx* = 2 mm *dy* = 5 mm	*dx* = 2 mm *dy* = 5 mm
FWHM Whole reconstruction	*dx* = 4.4 mm *dy* = 6 mm	*dx* = 11.8 mm *dy* = 10 mm
FWHM US Guided reconstruction	*dx* = 1.8 mm *dy* = 4 mm	*dx* = 4.5 mm *dy* = 6 mm

**Fig. 10:**
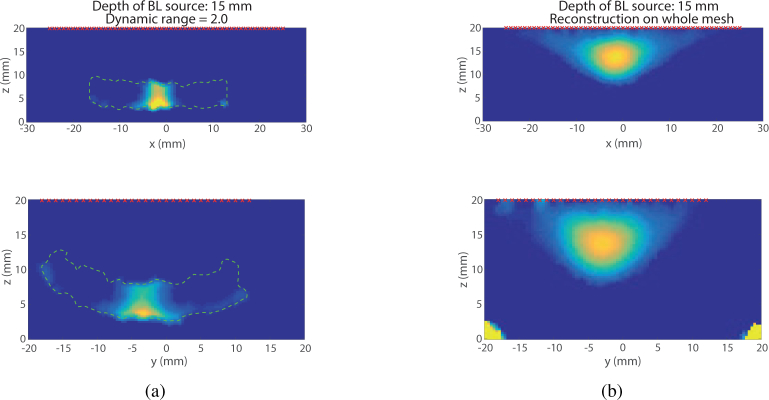
Visual comparison of two reconstruction methods (US Guided PR (a) and Whole mesh (b)) for a source 15.5 mm deep in the experimental phantom. (Top) XZ Plane, (Bottom) XY Plane.

. In both cases, shallow source and deep source, the error on the estimation of the source size is much smaller using the US guided permissible region than when reconstructing using the whole mesh. In addition, it is worth noticing that in the case of the deep source (*d* = 15.5 mm), the reconstruction using the whole mesh recovers a source at a shallower depth than expected. In contrast, the source reconstructed using the permissible region (Fig. 10(a)

) is closer to the true position of the source as driven by the choice of the permissible region centred around the correct depth given by the ultrasound image.

## 5. Discussion

The proposed reconstruction method, defining a permissible region guided by ultrasound and driven by the reduction of the dynamic range of the sensitivity matrix, shows a more stable and quantitatively accurate recovery of a bioluminescent source in BLT. The numeric simulations (the ultrasound was simulated by the knowledge of the position of the source) showed an improvement of the quantitative accuracy of the 3D bioluminescent tomography of approximately 25% and 10% for slab model and the Digimouse model respectively. The reconstructions on experimental data from a phantom gel showed that using this strategy provides a more accurate localisation of a light source deep inside the tissue (deeper than a cm) than a simple reconstruction on the whole mesh. In addition, a significant improvement of the recovered size of the source has been observed.

The choice of the dynamic range is important since it controls the size of the reconstructed volume. In practice, it is not possible to determine an optimal dynamic range by estimating metrics such as VR, DICE and MSE, therefore the value of the dynamic range has to be chosen manually keeping in mind several considerations. The criterion used to choose the correct value can be formulated using several factors: choosing the smallest dynamic range so that the source is contained within the permissible region with a good confidence. The necessary dynamic range could be estimated using knowledge of the potential size of the source and an examination of the ultrasound images. However, in certain cases this approach of limiting the dynamic range of the system is not always possible. For example, when the source has an elongated shape that spans a region with a very large dynamic range, or if the sample presents multiple sources dispersed over a region with a large dynamic range.

Using ultrasound imaging as a guide for the reconstruction was motivated by several factors. First, ultrasound is commonly used for biomedical applications because of the high compatibility with *in vivo* experiment, its non-invasive nature and its high resolution. Indeed, small animal research does not require high penetration depth, therefore it is possible to use high frequency ultrasound (*f_US_* ⩾ 15 MHz). This frequency range yields sub-millimetre resolution, which is compatible with the detection of millimetre size inclusion such as tumours [24, 25]. Second, ultrasound equipment is relatively cheap and compact, which makes it easy to integrate in an optical setup. However, this proposed strategy is limited to bioluminescent studies involving sources having a contrast in ultrasound imaging or other related techniques such as elastography. This is a limitation which is not considered to be too restrictive since ultrasound can be used to observe most major organs and many different cases of tumours related studies [24, 25].

## 6. Conclusion

This study showed a significant improvement of the accuracy of the evaluation of the light level of an embedded source using BLT (up to 25% in certain cases). In practice, as the light level of bioluminescent sources is linked to the amount of tagged cells, improving the accuracy has a meaningful biological significance. It leads to a better evaluation of the number of monitored cells, which, in turn, leads to a refinement of biological processes monitoring and a reduction of the number of animals used. The interest of this strategy is in the definition of the permissible region. The reconstructed volume is defined based on physical characteristics of the sample – the position of the source given by ultrasound imaging –, and characteristics of the model – dynamic range of the sensitivity matrix to stabilise the reconstruction process. The combination of bioluminescence tomography with ultrasound imaging, has great potential for extension into a wide range of non-invasive small animal imaging studies. In the experimental results presented here, the two imaging processes were performed at different times. However, in future experiments, performing both imaging approaches simultaneously would be beneficial, especially during *in vivo* experiments. Data provided by ultrasound could be used to further improve the BLT reconstruction by adapting the digital model with size and location of different organs for example. Parallel multi-modal imaging of a rodent could be achieved by, for example, integrating the ultrasound probe directly inside the dark chamber under the animal platform. Using a small aperture in the platform and a coupling medium, the animal can be ultrasonically imaged through its front while the optical system is imaging its back. Since rodents are not bigger than a few centimetres, this configuration enables ultrasound imaging of whole section of the animal, while avoiding he rib cage, which could deteriorate image quality.

More generally, as many imaging techniques are now available it is crucial to investigate how to couple them to improve quantity and accuracy of the information about the studied system. This is especially true in the case of biological studies involving cohorts of animals in order to refine results while reducing animal use.
